# Lubricin Levels in Temporomandibular Joint Disorders: A Scoping Review

**DOI:** 10.3390/ijms27115035

**Published:** 2026-06-02

**Authors:** Paweł Sikora, Maciej Chęciński, Tomasz Horodniczy, Kamila Chęcińska, Natalia Turosz, Kalina Romańczyk, Amelia Hoppe, Maciej Sikora

**Affiliations:** 1 Faculty of Medicine, Medical University of Lublin, Al. Racławickie 1, 20-059 Lublin, Poland; pawelsikora2205@gmail.com; 2 National Medical Institute of the Ministry of the Interior and Administration, Wołoska 137, 02-507 Warsaw, Poland; maciej.checinski@pimmswia.gov.pl (M.C.); natalia.turosz@pimmswia.gov.pl (N.T.); maciej.sikora@pimmswia.gov.pl (M.S.); 3 Department of Maxillofacial Surgery, Hospital of the Ministry of the Interior and Administration, Wojska Polskiego 51, 25-375 Kielce, Poland; 4 ORTHO.PL Ortodonta Zdrowego Uśmiechu Dzieci i Dorosłych Wrocław, Buforowa 34, 52-131 Wroclaw, Poland; tomasz.horodniczy2@gmail.com; 5 Department of Oral Surgery, Preventive Medicine Center, Komorowskiego 12, 30-106 Cracow, Poland; kalina.romanczyk@wp.pl (K.R.); amelia.a.hoppe@gmail.com (A.H.); 6 Department of Biochemistry and Medical Chemistry, Pomeranian Medical University, Powstańców Wielkopolskich 72, 70-111 Szczecin, Poland

**Keywords:** lubricin, proteoglycan 4, *PRG4*, temporomandibular joint, temporomandibular disorders, synovial fluid, osteoarthritis, scoping review

## Abstract

Lubricin, also known as proteoglycan 4 (*PRG4*), is a key glycoprotein involved in boundary lubrication and maintenance of joint homeostasis in the temporomandibular joint (TMJ). However, the clinical evidence regarding synovial fluid (SF) lubricin levels remains limited and fragmented. This scoping review aimed to map and synthesize the available clinical evidence on lubricin levels in patients with temporomandibular disorders (TMDs) and their relationship to clinical outcomes, particularly pain and mandibular mobility. Searches were conducted in PubMed, Scopus, ACM, BASE, Cochrane, ClinicalTrials.gov, and Google Scholar. After duplicate removal and screening, two studies were included in the final synthesis. Preliminary findings from these studies suggest that (SF) lubricin levels may be lower in more advanced TMJ pathology, particularly in degenerative disease. Earlier stages of internal derangement showed lubricin concentrations closer to those observed in healthy controls, whereas advanced internal derangement and osteoarthritic disease were potentially associated with lower levels. One study also reported an inverse correlation between lubricin concentration and pain intensity, while the other demonstrated impaired boundary lubrication in TMD groups. Overall, the available clinical evidence is very limited and insufficient to establish *PRG4* as a validated biomarker but suggests a possible association between reduced SF lubricin levels and more advanced TMJ disease. Further well-designed clinical studies are required to confirm these observations and clarify their diagnostic and therapeutic relevance.

## 1. Introduction

### 1.1. Rationale

Lubricin, also known as proteoglycan 4 (*PRG4*), is a high-molecular-weight glycoprotein belonging to the mucin-like protein family that plays a crucial role in maintaining the integrity and homeostasis of joint surfaces. Under physiological conditions, it is synthesized primarily by fibroblast-like synovial cells (type B synoviocytes) and by chondrocytes of the superficial layer of joint cartilage (superficial zone chondrocytes). The physiological presence of lubricin is not limited to the TMJ; it is a fundamental component of the synovial fluid in major weight-bearing joints, such as the knee and hip, and has been identified in menisci, tendons, and the periodontal ligament [1]. Studies focusing on anterior cruciate ligament (ACL) injuries have demonstrated that a significant reduction in synovial lubricin concentrations compromises boundary lubrication. This deficiency increases the risk of wear-induced damage and serves as a key factor in the pathogenesis of osteoarthritis [2]. In the temporomandibular joint (TMJ), *PRG4* expression is found in both the synovium and the condylar cartilage and joint disc, indicating its important role in maintaining the proper biomechanical properties of this joint [3]. *PRG4* gene expression has been shown to be regulated by mechanical stress and inflammatory mediators, while its downregulation accompanies degenerative and post-traumatic changes, leading to increased friction and progressive cartilage degradation [4].

**Figure 1:**
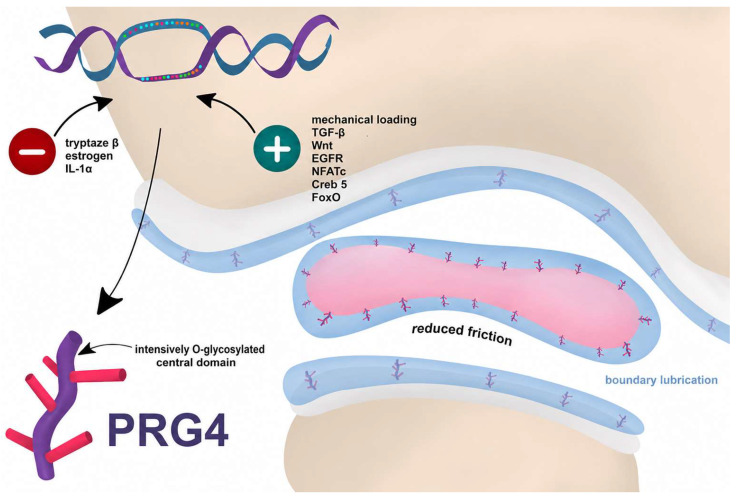
Regulatory factors of *PRG4* expression and its function. *PRG4* expression is regulated by both mechanical stimuli and molecular mediators, including cytokines, growth factors, and transcription factors. In a healthy TMJ, the hydrophilic, intensively O-glycosylated central domain of *PRG4* enables the formation of a stable boundary layer, permanently bound to the cartilage, reducing friction. Original illustration created by the co-authors (A.H).

Structurally, lubricin contains an extensive, intensively O-glycosylated central domain, which gives it marked hydrophilic properties and a negative electrostatic charge (Figure 1) [4,5,6]. These properties enable protein adhesion to the cartilage surface and the formation of a thin, stable boundary layer with a very low coefficient of friction (COF). Lubricin is present both in the fluid phase of synovial fluid (SF) and as a surface layer permanently bound to cartilage [7]. Its presence enables so-called boundary lubrication, which is crucial under high loads and low movement speeds, when hydrodynamic lubrication mechanisms become insufficient [8]. In this way, *PRG4* reduces mechanical wear and protects the extracellular matrix from damage [9].

The function of lubricin is complementary to that of hyaluronic acid (HA), which is primarily responsible for maintaining SF viscosity and hydrodynamic lubrication, ensuring the separation of joint surfaces. Both components work together to ensure continuity of the lubrication process under various biomechanical conditions [8]. In vitro studies have also shown that HA can modulate *PRG4* expression in synovial cells, which partially explains the protective and anti-inflammatory effects observed after intra-articular HA injections [10]. Available clinical evidence suggests that reduced lubricin concentrations in TMJ SF are observed primarily in advanced or degenerative joint pathology rather than uniformly across all temporomandibular joint disorders. This suggests that *PRG4* may be involved not only in joint lubrication but also in the progression of degenerative changes within the TMJ [11,12].

Advances in protein engineering have enabled the development of a recombinant form of lubricin exhibiting properties similar to the endogenous protein [13]. In preclinical studies conducted in animal models, including experimental models of TMJ degeneration, recombinant human *PRG4* administration reduced friction, extracellular matrix degradation, and inflammation while improving joint function [14,15]. However, there are currently no published clinical studies evaluating the efficacy and safety of intra-articular *PRG4* injections in humans, and the protein remains in the experimental phase [16,17,18].

The available literature on lubricin in temporomandibular joint disorders (TMDs) appears sparse and heterogeneous, with limited clinical evidence and variable outcome reporting. Therefore, a scoping review approach was chosen to map the available evidence, characterize the existing studies, and identify knowledge gaps rather than to perform a quantitative effect estimate.

### 1.2. Objectives

The objective of this scoping review was to map and synthesize the available clinical evidence on SF lubricin levels in patients with TMDs and their relationship to available clinical outcomes, particularly pain and mandibular mobility.

## 2. Materials and Methods

### 2.1. Protocol and Registration

The scoping review was performed and reported following the PRISMA-ScR guidelines and checklist. A review protocol was developed prior to study initiation and registered in the OSF repository under registration number osf.io/u4tme.

### 2.2. Eligibility Criteria

The PICOTS framework was adapted to structure the eligibility criteria. The study population was defined as patients with diagnosed TMDs. Specific diagnoses were taken into account, but they did not affect the eligibility of the report for review. Studies on human cadavers and any animal tissues were excluded. Eligible studies were required to report SF lubricin assessment. Possible viscosupplementation was permissible. The outcomes of interest included SF lubricin level and, where available, clinical variables such as health-related quality of life, joint pain, and mandibular mobility. There was no time limit for the publication of the source reports, but unpublished papers, i.e., preprints, were not accepted. Studies conducted in human participants with TMDs were eligible, regardless of whether they used an observational or interventional design. Due to insufficient rigor, reports other than articles in scientific journals, e.g., conference proceedings, were rejected. No language restrictions were applied. The abbreviated criteria for qualifying reports for the review are summarized in Table 1.

### 2.3. Information Sources

Several methods were utilized to search the medical literature for eligible reports (T.H. and M.C.). We used multiple bibliographic databases and scholarly search engines, including PubMed, Scopus, ACM, Bielefeld Academic Search Engine (BASE), and Cochrane, to search for indexed records. The ACM Digital Library was included to capture interdisciplinary records related to lubricin, boundary lubrication, biomaterials, tribology, bioengineering, and biomimetic or synthetic cartilage lubricants, which may not be indexed in strictly biomedical databases. Next, we searched ClinicalTrials.gov for registered trials and references to relevant publications. In the third step, we performed the gray literature search with the Google Scholar search engine [19,20,21,22,23,24,25].

### 2.4. Search

Searches for primary research reports were conducted primarily in medical databases. The query was formulated based on the eligibility criteria specified above. It was refined during preliminary searches. The following query was finally used in all search engines: “(lubricin OR “proteoglycan 4” OR proteoglycan4 OR proteoglycan-4 OR “prg 4” OR prg4 OR prg-4 OR camptodactyly OR cacp OR “joint capsule lubricating protein” OR jcap OR “megakaryocyte stimulating factor” OR msf OR “superficial zone protein” OR szp OR szp1 OR bg174l6.2 OR lubricin-mimetic OR lubricin-mimic OR mlub OR “bio-inspired lubricant” OR “biomimetic lubricant” OR “synthetic cartilage lubricant”) AND (temporomandibular OR tmj OR tmd)”. In the case of the Google Scholar search engine, we used the preceding command ‘Allintitle’ to adhere to specified keywords. Main searches were performed on 13 January 2025 and updated on 22 February 2026.

### 2.5. Selection of Sources of Evidence

Identified records were entered into the Rayyan automation tool (version 2024.08.29; Rayyan Systems Inc., Cambridge, MA, USA), which identified potential duplicates. Manual deduplication was performed by one of the authors (M.C.). Two investigators (P.S. and M.C.) performed independent blinded selection, which consisted of (1) screening and (2) eligibility stages. The first stage involved screening titles and abstracts against the predefined eligibility criteria. The second stage differed in the assessment of full-text reports. Records were advanced to full-text assessment if at least one reviewer considered them potentially eligible. In case of disagreement on the final inclusion of a report, a simple majority open vote was held with the participation of the two previous assessors (M.C. and P.S.) and a third investigator (K.C.). Exclusion of a report at the full-text evaluation stage involved citing it in this review and providing a reason for rejection.

### 2.6. Data Charting Process

Data extraction from the source reports was performed by a single investigator (P.S.) and verified by a second one (M.C.). The entire data collection was conducted without the use of an automation tool and consisted of transferring selected elements to the appropriate columns in previously prepared tables. It was permissible to add rows corresponding to subsequent groups of patients and columns specifying the length of post-intervention observation. A predesigned data-charting table was used. No contact with study authors was undertaken to obtain missing data.

### 2.7. Data Items

Data items were grouped into three categories: (1) study characteristics, (2) patient group characteristics, and (3) outcome variables. Study characteristics included the first author, year of publication, total number of participants, and any interventions reported in the study. Patient group characteristics included the diagnosis (or confirmation that the group consisted of healthy controls without TMD), number of participants, and number of affected joints.

The outcomes of interest included SF lubricin level and, where available, clinical variables such as health-related quality of life, joint pain intensity, and mandibular mobility. Data were extracted for baseline measurements and, where applicable, for follow-up periods.

SF lubricin concentration was recorded in nanograms per milliliter (ng/mL). Any health-related quality-of-life scale was eligible; when reported, these values were converted to integer percentages of the maximum possible score. For pain assessment, spontaneous pain was preferred. If such data were unavailable, retrospectively reported pain was extracted; if this was also unavailable, provoked pain intensity was used. Pain values were converted to a 0–10 scale and reported to one decimal place.

Mandibular mobility was preferably extracted as unassisted maximum mouth opening (MMO) measured between the incisal edges of natural teeth or restorations. If this variable was not reported, assisted MMO, MMO without pain, protrusive movement, or, as a last resort, average lateral movement was extracted. Mandibular mobility values were converted to millimeters (mm) and reported to one decimal place.

### 2.8. Critical Appraisal of Individual Sources of Evidence

To enhance data interpretability, a narrative critical appraisal of the included studies was conducted based on the core domains of the AXIS (Appraisal tool for Cross-Sectional Studies) tool [26].

### 2.9. Synthesis of Results

The charted data were synthesized descriptively and narratively, with emphasis on study characteristics, lubricin concentrations, and their reported associations with clinical variables such as pain and mandibular mobility.

## 3. Results and Discussion

### 3.1. Selection of Sources of Evidence

The literature search identified a total of 228 records for screening across PubMed, ACM, BASE, Scopus, and Google Scholar. Of these, 47 records were retrieved from PubMed, 29 from ACM, 78 from BASE, 51 from Scopus, and 23 from Google Scholar [19,20,21,22,23,24,25]. Additional searches of ClinicalTrials.gov and the Cochrane Library did not identify any relevant records (Table A1).

After importing the 228 records from the main bibliographic databases, 122 duplicates were removed, leaving 106 unique records for title and abstract screening. At this stage, 97 records were excluded by both reviewers. The remaining 9 records were considered potentially eligible and were assessed in full text. Of these, 2 studies had been concordantly judged as potentially relevant during title and abstract screening, and another 7 studies progressed to full-text assessment following disagreement at the screening stage.

**Figure 2:**
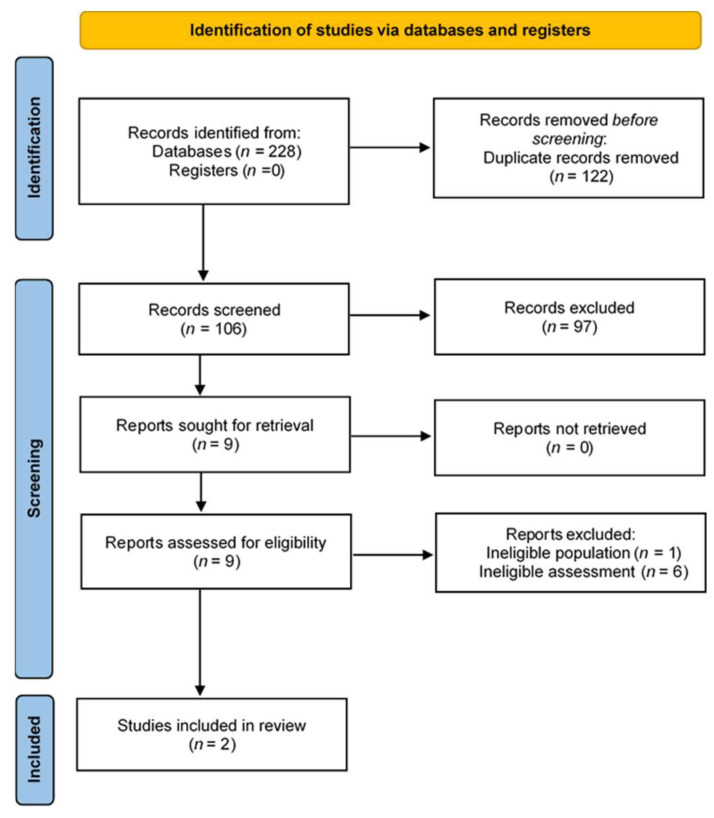
Flow diagram of study selection.

Following full-text review, 7 articles were excluded, and 2 studies were ultimately included in the synthesis (Figure 2). Reasons for exclusion were assigned according to the first eligibility domain that was not met (Table A2).

### 3.2. Characteristics of Sources of Evidence

Two studies were included in the final synthesis: Leonardi et al. (2016) [11], entitled “Lubricin in synovial fluid of mild and severe temporomandibular joint internal derangements”, and Wei et al. (2010) [12], entitled “Boundary-lubricating ability and lubricin in synovial fluid of patients with temporomandibular joint disorders”. Both studies focused on lubricin in the SF of patients with TMDs and addressed the role of lubricin in the context of TMJ (Table 2) [11,12].

### 3.3. Critical Appraisal Within Sources of Evidence

Both studies demonstrate a moderate level of methodological quality, with clear aims, appropriate study design, and well-described, validated outcome measures. However, they are limited by several factors, including a lack of justification for sample size, unclear representativeness of the sampling process, and absence of non-response bias assessment (Table 3).

### 3.4. Results of Individual Sources of Evidence

Leonardi et al. (2016) [11] evaluated lubricin levels in SF in relation to the severity of TMJ internal derangements according to the Wilkes classification. The study included 34 TMJs from patients with Wilkes stages III, IV, and V and eight control joints obtained from patients undergoing orthognathic surgery without TMJ pathology. Lubricin concentration was determined using an enzyme-linked immunosorbent assay (ELISA). The authors reported a progressive decrease in lubricin levels with increasing disease severity. Mean lubricin concentrations were 7425.0 ± 340.0 ng/mL in the control group, 7029.0 ± 210.0 ng/mL in stage III, 5640.0 ± 100.0 ng/mL in stage IV, and 4780.0 ± 110.0 ng/mL in stage V. Lubricin levels in stages IV and V were significantly lower compared with controls. Furthermore, lubricin concentration showed a significant inverse correlation with patient age and pain intensity measured using the visual analogue scale, indicating that lower lubricin levels were associated with older age and higher pain intensity, while no correlation was found with maximal interincisal opening (MIO) [11].

Wei et al. (2010) [12] investigated lubricin concentration and boundary lubrication ability in SF collected from patients with different stages of TMD, including disc displacement with reduction (DDR), disc displacement without reduction (DDNR), and osteoarthritis (OA). The study included 34 patients with TMD and seven healthy controls. Lubricin concentrations were measured using an enzyme-linked immunosorbent assay (ELISA). The authors reported lubricin levels of 7496.0 ± 468.0 ng/mL in healthy controls, 7160.0 ± 1249.0 ng/mL in the DDR group, and 7215.0 ± 1117.0 ng/mL in the DDNR group. Significantly lower concentrations were observed in patients with OA (5689.0 ± 1313.0 ng/mL). These findings indicate that lubricin levels were not clearly reduced in earlier non-degenerative stages of TMD, such as disc displacement with or without reduction, but were lower in the presence of osteoarthritic joint changes. Additionally, the COF, reflecting boundary lubrication capacity, was significantly higher in all TMD groups compared with controls, suggesting impaired lubrication of the joint. However, no significant correlation was found between lubricin concentration and the COF [12].

Overall, both studies reported lower lubricin levels in SF in more advanced or degenerative TMJ pathology. In contrast, earlier stages of internal derangement or non-degenerative disc displacement showed lubricin concentrations closer to those observed in healthy controls [11,12].

### 3.5. Synthesis of Results

This scoping review identified a very limited body of clinical evidence addressing lubricin in TMDs. Although the search across the main bibliographic databases retrieved 228 records, only two studies fulfilled the eligibility criteria after duplicate removal, title and abstract screening, and full-text assessment. This marked reduction highlights both the narrow scope of the available literature and the scarcity of studies directly examining lubricin in relation to TMJ pathology.

Direct comparisons of absolute lubricin concentrations between the included studies are precluded by significant methodological differences, including differences in diagnostic classification systems, synovial fluid centrifugation protocols, and commercial ELISA kits. The two included studies were consistent in suggesting that lubricin levels in SF are lower in more advanced or degenerative TMJ disease, whereas earlier non-degenerative stages showed values closer to those of healthy controls. In contrast, earlier stages of internal derangement appeared to be associated with lubricin concentrations that were closer to those observed in healthy controls; specifically, a minor 4.5% reduction in DDR and 3.7% in DDNR was observed by Wei et al. (2010) [12], while Leonardi et al. (2016) [11] reported a 5.3% reduction in Wilkes stage III. Taken together, these findings suggest that alterations in lubricin may be linked more strongly to disease progression and joint degeneration than to the earliest functional stages of TMDs, as evidenced by a significant 24.1% reduction in the osteoarthritis group in the former study and profound reductions of 24.0% in Wilkes stage IV and 35.6% in Wilkes stage V in the latter [11,12].

At the same time, the identified evidence remains limited not only in quantity but also in scope. Both included studies focused on SF measurements, and neither provided sufficient evidence to establish lubricin as a validated clinical biomarker or therapeutic target. Moreover, the broader body of literature retrieved during the review frequently proved ineligible because studies were preclinical, lacked clinically relevant patient outcomes, or did not assess lubricin directly. This pattern indicates that, despite growing interest in biological mechanisms related to joint lubrication, the clinical literature specifically addressing lubricin in TMDs remains underdeveloped [11,12].

Overall, the available evidence suggests that lubricin is a potentially relevant biological component in the pathophysiology of TMDs, particularly in relation to degenerative changes and impaired joint lubrication. However, the current evidence base is too limited to support firm conclusions regarding its diagnostic, prognostic, or therapeutic significance. Further clinical studies are needed to clarify the relationship between lubricin concentration, disease stage, pain, joint function, and structural joint changes.

### 3.6. Summary of Evidence

The present analysis evaluated the available evidence regarding lubricin concentration in the SF of patients with TMD. Both included studies demonstrated that lubricin levels are associated with the severity of TMJ pathology, suggesting that this glycoprotein plays an important role in maintaining joint homeostasis and protecting articular cartilage from mechanical damage.

Wei et al. (2010) [12] reported that lubricin concentrations in SF were similar between healthy controls and patients with early stages of TMDs, including DDR and DDNR. However, a significant decrease in lubricin levels was observed in patients with OA. These findings suggest that the reduction in lubricin is mainly associated with degenerative changes in the joint rather than with early internal derangements. The authors also demonstrated impaired boundary lubrication in all TMD groups, as indicated by a higher COF compared with healthy individuals. This observation indicates that alterations in the lubricating properties of SF may occur even before significant changes in lubricin concentration become detectable [12].

Similarly, Leonardi et al. (2016) [11] demonstrated a progressive decrease in lubricin levels with increasing severity of TMJ internal derangements according to the Wilkes classification. While lubricin concentrations in stage III patients were comparable to those observed in the control group, significantly lower levels were found in stages IV and V. These results are consistent with the interpretation that more advanced TMJ pathology is accompanied by lower lubricin levels, particularly in later stages of internal derangement. Moreover, the study revealed a significant inverse correlation between lubricin concentration and pain intensity, hypothetically suggesting that lower lubricin levels may be associated with greater inflammatory burden and mechanical stress within the TMJ, although these specific variables were not directly quantified in the primary research [11].

While the reduction in lubricin observed in advanced stages of TMDs may reflect both inflammatory and mechanical processes, it must be explicitly clarified that these mechanistic interpretations are inferred from the broader literature on other articulating joints and were not directly demonstrated by the included TMJ studies. Previous studies in general joint models have shown that pro-inflammatory cytokines, such as interleukin-1β and tumor necrosis factor-α, can downregulate lubricin synthesis and increase its degradation within the joint environment [27]. In addition, mechanical overloading of the joint is hypothesized to impair lubricin production by synovial fibroblasts and chondrocytes [28]. Together, these mechanisms may be involved in the disruption of the protective lubrication system of the joint, thereby promoting increased friction, cartilage wear, and progression of degenerative changes [27,28].

**Figure 3:**
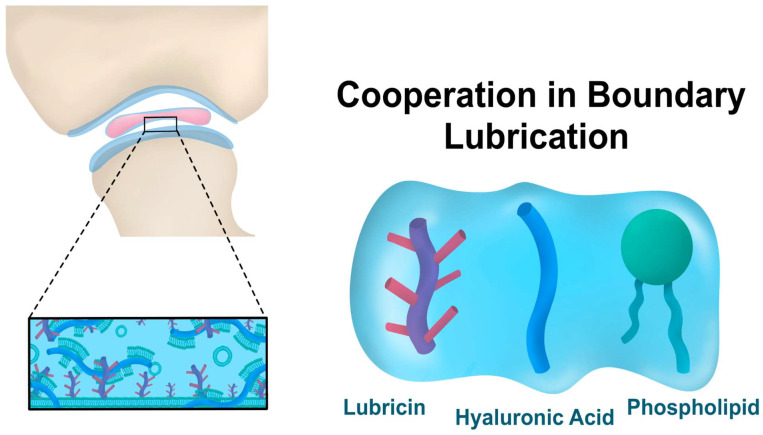
Synergistic molecular cooperation in maintaining a stable boundary layer. Proper joint mobility relies on the effective lubrication of articular cartilage, which is maintained mainly by the synergistic interaction of three molecules: hyaluronic acid anchored by the lubricin bound to the cartilage and surface-active phospholipids. Original illustration created by one of the co-authors (A.H.).

Another important aspect highlighted by the analyzed studies is the multifactorial nature of joint lubrication. Although lubricin plays a critical role in boundary lubrication, the lubricating properties of SF also depend on other molecules, particularly HA and various synovial proteins (Figure 3) [29]. Therefore, alterations in joint lubrication observed in patients with TMD may result from complex biochemical interactions within the SF rather than from changes in lubricin concentration alone [11,12].

From a clinical perspective, the observed association between decreased lubricin levels and advanced stages of TMDs suggests possible therapeutic implications. Restoration of joint lubrication through intra-articular administration of recombinant lubricin or agents stimulating its production may represent a theoretical strategy for limiting cartilage degeneration and improving joint function. However, since current evidence is limited to observational findings, further clinical trials are required before the clinical utility of lubricin-based treatments can be established in humans.

### 3.7. Strengths

One of the main strengths of this review is its focused scope, which allowed for a targeted evaluation of the available evidence regarding lubricin in the SF of patients with TMDs. By concentrating specifically on clinical studies assessing lubricin in relation to TMJ pathology, this review addresses a narrowly defined yet clinically relevant research question. Another important strength is the breadth of the search strategy. Several major bibliographic databases and scholarly search engines were searched, including PubMed, Scopus, ACM, BASE, ClinicalTrials.gov, Cochrane, and Google Scholar, which increased the likelihood that relevant evidence was identified. A further strength is the structured and transparent selection process, conducted in accordance with the PRISMA-ScR framework [30], which improves reproducibility and enables clear reporting of study identification, screening, eligibility assessment, and inclusion. In addition, the synthesis highlights not only the scarcity of the available evidence but also that both studies demonstrated similar trends, although these preliminary findings remain methodologically limited.

### 3.8. Limitations

Despite these findings, several limitations should be considered. First, the number of available studies investigating lubricin in the TMJ remains very limited, and the included studies were based on relatively small patient populations. In addition, differences in SF collection methods and analytical techniques may have influenced the reported lubricin concentrations and reduced comparability across studies.

Another important limitation is the heterogeneity of the included clinical populations and disease classifications. Although both studies addressed TMDs, they used somewhat different clinical groupings, including internal derangement, disc displacement, and OA. This heterogeneity makes direct comparison difficult and limits the ability to define precise threshold values or stage-specific patterns of lubricin reduction. Crucially, potential confounding variables such as age, sex, baseline inflammation, concurrent medications, and disease duration were not consistently controlled for across the primary studies. These unmeasured factors might independently influence lubricin concentrations, further complicating the interpretation of the results.

Furthermore, because only two studies were eligible for inclusion, the present review was not able to statistically explore or rule out potential publication bias or perform subgroup comparisons. Importantly, the reliance on cross-sectional design studies with a lack of longitudinal data means we cannot draw robust conclusions regarding causality. Additionally, the absence of a formal risk-of-bias assessment further complicates the interpretation of the results. The scoping nature of the review should also be emphasized, as its purpose was to map the available evidence rather than to provide a formal quantitative synthesis or pooled effect estimate.

Finally, although multiple bibliographic databases and scholarly search engines were searched, it remains possible that some relevant studies indexed exclusively in other sources were not identified. Taken together, these limitations indicate that the current evidence base should be interpreted cautiously and that further research involving larger cohorts and standardized methodologies is needed to clarify the role of lubricin in the pathogenesis of TMDs.

### 3.9. Implications

The findings of this review suggest that lubricin may be a potentially relevant biological marker of disease progression in TMDs, particularly in the context of degenerative joint changes. If confirmed in future studies, lubricin assessment in SF could contribute to a better understanding of disease severity and to the identification of patients at greater risk of progressive joint deterioration. The observed relationship between lower lubricin levels, advanced pathological changes, and pain intensity also supports the concept that impaired boundary lubrication may be one of the mechanisms contributing to symptom progression and structural joint damage [30].

These findings may also have therapeutic implications. Interventions aimed at restoring synovial lubrication, whether through exogenous lubricin supplementation, stimulation of endogenous lubricin production, or broader modulation of the synovial environment, may represent a promising direction for future treatment development [31]. However, at present, the evidence is insufficient to justify clinical application, and lubricin should still be considered an experimental target rather than an established diagnostic or therapeutic tool in TMDs.

### 3.10. Future Research

Future studies should focus on larger and better-characterized patient cohorts, with clear diagnostic criteria and consistent staging systems for TMDs. Standardization of SF sampling procedures, laboratory methods for lubricin quantification, and reporting of clinical variables would substantially improve comparability across studies. In particular, future research should investigate whether lubricin levels correlate not only with disease stage and pain intensity but also with imaging findings, inflammatory markers, mandibular function, and longitudinal clinical outcomes.

Prospective studies are especially needed to determine whether reduced lubricin levels precede structural degeneration or arise as a consequence of ongoing joint damage. Such studies would help clarify whether lubricin has value as an early biomarker, a marker of progression, or both. Further research should also examine the interactions between lubricin and other components of SF, especially HA, inflammatory mediators, and proteins involved in cartilage metabolism. Finally, experimental and translational studies evaluating lubricin-based therapies may help determine whether modulation of joint lubrication can alter the course of TMJ degeneration.

The presented findings indicate the therapeutic potential of exogenous lubricin. Despite these promising observations, there are no published clinical studies evaluating the efficacy and safety of intra-articular *PRG4* injections in humans [14]. These limitations stem primarily from the difficulty of producing stable, biologically active forms of recombinant lubricin; the high cost of its production; and the lack of data on the immunogenicity and persistence of the protein in the joint cavity [16,17]. Unlike widely used and clinically approved HA preparations, *PRG4* is currently in the experimental phase, but preclinical findings clearly indicate its substantial potential in the treatment of functional and degenerative disorders of the TMJ [18].

## 4. Conclusions

The available clinical evidence suggests that lubricin concentration in SF is reduced in more advanced stages of TMDs, particularly in the presence of degenerative changes. Both included studies indicate that lubricin may play an important role in maintaining joint lubrication and protecting the TMJ from progressive degenerative changes, potentially by mitigating mechanical and inflammatory stress. At the same time, the current evidence base remains very limited and does not permit definitive conclusions regarding the diagnostic or therapeutic utility of lubricin.

Overall, lubricin represents a biologically plausible but experimental candidate biomarker in the context of TMJ pathophysiology, whose clinical significance remains uncertain and requires confirmation through larger, prospective studies.

## Figures and Tables

**Table 1:** Eligibility criteria.

Domain	Criteria for Inclusion	Criteria for Exclusion
Population	Patients diagnosed with TMD	Human cadavers or any animal tissues
Assessment	Synovial-fluid lubricin assessment	None
Comparison	Healthy controls or none	None
Outcomes	Lubricin levels, health-related quality of life, joint pain, or mandibular mobility	Unquantifiable results
Timeframe	Any	Unpublished reports
Settings	Clinical studies in humans	Non-clinical studies, non-journal reports

**Table 2:** Characteristics of sources of evidence.

First Author, Year	Study Design	Participants (*n*)	Joints (*n*)	Diagnosis Groups	Control Group	Assay Characteristic	Outcomes	Main Findings	Synovial Fluid Collection Procedures	Exclusion Criteria	Statistical Approaches
Leonardi et al., 2016 [11]	Cross-sectional study	32 patients, mean age: 34 ± 11.04 (27 females + 5 males) + controls, mean age: 42.3 ± 9.5 (6 females + 2 males)	34 TMD joints + 8 control joints	Wilkes stage III, IV, V	Yes (orthognathic patients without TMJ pathology)	ELISA (Pierce Biotechnology, Waltham, MA, USA); sensitivity: 3.25 pg/mL; calculated per mg of total protein.	Lubricin (ng/mL): control 7425 ± 340; stage III 7029 ± 210; stage IV 5640 ± 100; stage V 4780 ± 110; correlations with age, VAS, Maximum Interincisal Opening (MIO)	Progressive decrease with severity; significantly lower in IV–V vs. control; inverse correlation with age and VAS; no correlation with MIO	Aspiration with dilution (1.0 mL saline) during arthrocentesis; 21G needle; “push-and-pull” technique	a diagnosis of Wilkes stage I or II, systemic arthropathy, use of nonsteroidal anti-inflammatory drugs, and/ or history of trauma	Shapiro–Wilk, Mann–Whitney U test, Spearman correlation
Wei et al., 2010 [12]	Cross-sectional study	34 TMD patients, mean age: 31 (29 females + 5 males) + 7 controls, mean age: 27 (4 females + 3 males)	Not reported	DDR, DDNR, OA	Yes (healthy controls)	ELISA (Yope Biotechnology, Shanghai, China); total protein quantified via Micro BCA assay (Pierce Biotechnology, Waltham, MA, USA)	Lubricin (ng/mL): control 7496 ± 468; DDR 7160 ± 1249; DDNR 7215 ± 1117; OA 5689 ± 1313; COF: control 0.247 ± 0.006; DDR 0.266 ± 0.011; DDNR 0.268 ± 0.010; OA 0.275 ± 0.009	Lubricin reduced only in OA; COF higher in all TMD groups; no correlation between lubricin and COF	Aspiration with dilution (1.0 mL saline) during local anesthesia; superior joint compartment; 5-fold “push-and-pull” technique	Prior TMJ treatment; any medication (including NSAIDs) within 2 weeks prior to synovial fluid sampling	1-way analysis of variance test, Bonferroni test and correlation coefficient calculation

**Table 3:** Critical appraisal of the included studies.

	Leonardi et al. [11]	Wei et al. [12]
Introduction		
1. Were the aims/objectives of the study clear?	Yes	Yes
Methods		
2. Was the study design appropriate for the stated aim(s)?	Yes	Yes
3. Was the sample size justified?	No	No
4. Was the target/reference population clearly defined? (Is it clear who the research was about?)	Yes	Yes
5. Was the sample frame taken from an appropriate population base so that it closely represented the target/reference population under investigation?	Unclear	Unclear
6. Was the selection process likely to select subjects/participants that were representative of the target/reference population under investigation?	Unclear	Unclear
7. Were measures undertaken to address and categorize non-responders?	No	No
8. Were the risk factor and outcome variables measured appropriate for the aims of the study?	Yes	Yes
9. Were the risk factor and outcome variables measured correctly using instruments/ measurements that had been trialed, piloted or published previously?	Yes	Yes
10. Is it clear what was used to determine statistical significance and/or precision estimates? (e.g., *p* values, CIs)	Yes	Yes
11. Were the methods (including statistical methods) sufficiently described to enable them to be repeated?	Yes	Yes
Results		
12. Were the basic data adequately described?	Yes	Yes
13. Does the response rate raise concerns about non-response bias?	Unclear	Unclear
14. If appropriate, was information about non-responders described?	No	No
15. Were the results internally consistent?	Yes	Yes
16. Were the results presented for the analyses described in the methods?	Yes	Yes
Discussion		
17. Were the authors’ discussions and conclusions justified by the results?	Yes	Yes
18. Were the limitations of the study discussed?	No	Yes
Other		
19. Were there any funding sources or conflicts of interest that may affect the authors’ interpretation of the results?	Yes	Yes
20. Was ethical approval or consent of participants attained?	Yes	Yes

## Data Availability

No new data were created or analyzed in this study. Data sharing is not applicable to this article.

## Abbreviations

The following abbreviations are used in this manuscript:

ACM	Association for Computing Machinery
BASE	Bielefeld Academic Search Engine
COF	Coefficient of Friction
DDNR	Disc Displacement without Reduction
DDR	Disc Displacement with Reduction
ELISA	Enzyme-Linked Immunosorbent Assay
HA	Hyaluronic Acid
MIO	Maximal Interincisal Opening
mL	Milliliter
mm	Millimeters
MMO	Maximum Mouth Opening
ng	Nanogram
OA	Osteoarthritis
OSF	Open Science Framework
PICOTS	Population, Intervention, Comparison, Outcomes, Timeframe, Study Design
*PRG4*	Proteoglycan 4
PRISMA	Preferred Reporting Items for Systematic Reviews and Meta-Analyses
SF	Synovial Fluid
TMD/TMDs	Temporomandibular Joint Disorders
TMJ	Temporomandibular Joint

## Appendix A

**Table A1:** Search results.

Search Engine	Total Available Records	Records Identified by Search
ACM [19]	3,798,395	29
BASE [20]	404,802,253	78
Clinicaltrials.gov [21]	517,703	0
Cochrane [22]	2,203,247	0
Google Scholar [23]	389,000,000	23
PubMed [24]	37,000,000	47
Scopus [25]	97,300,000	51

**Table A2:** Reports rejected on full-text evaluation.

First Author, Publication Year	Title	Reason for Exclusion
Kao, 2024 [32]	Chondroid Synoviocytic Neoplasm: A Clinicopathologic, Immunohistochemical, and Molecular Genetic Study of a Distinctive Tumor of Synoviocytes	Ineligible population
Qadri, 2023 [33]	Targeting CD44 Receptor Pathways in Degenerative Joint Diseases: Involvement of Proteoglycan-4 (*PRG4*)	Ineligible assessment
Balenton, 2018 [34]	Lubricin: Toward a Molecular Mechanism for Temporomandibular Joint Disorders	Ineligible assessment
Cen, 2018 [35]	Glucosamine oral administration as an adjunct to hyaluronic acid injection in treating temporomandibular joint osteoarthritis	Ineligible assessment
Leonardi, 2011 [36]	Lubricin immunohistochemical expression in human temporomandibular joint disc with internal derangement	Ineligible assessment
Tang, 2010 [37]	Effects of intra-articular administration of sodium hyaluronate on plasminogen activator system in temporomandibular joints with osteoarthritis	Ineligible assessment
Nitzan, 2003 [38]	‘Friction and Adhesive Forces’ Possible Underlying Causes for Temporomandibular Joint Internal Derangement	Ineligible assessment
